# ROS-Responsive and pH-Sensitive Aminothiols Dual-Prodrug for Radiation Enteritis

**DOI:** 10.3390/antiox11112145

**Published:** 2022-10-29

**Authors:** Yuanfang Chen, Yuwei Yang, Haikang Tang, Ziqi Zhang, Xiaoliang Zhou, Wenqing Xu

**Affiliations:** Institute of Radiation Medicine, Chinese Academy of Medical Sciences & Peking Union Medical College, Tianjin 300192, China

**Keywords:** ROS response, aminothiols, oral administration, radioprotection, intestinal damage

## Abstract

Radiation exposure can immediately trigger a burst of reactive oxygen species (ROS), which can induce severe cell death and long-term tissue damage. Therefore, instantaneous release of sufficient radioprotective drugs is vital to neutralize those accumulated ROS in IR-exposed areas. To achieve this goal, we designed, synthesized, and evaluated a novel oral ROS-responsive radioprotective compound (M1) with high biocompatibility and efficient ROS-scavenging ability to act as a promising oral drug for radiation protection. The compound is stably present in acidic environments and is hydrolyzed in the intestine to form active molecules rich in thiols. M1 can significantly remove cellular ROS and reduce DNA damage induced by γ-ray radiation. An in vivo experiment showed that oral administration of M1 effectively alleviates acute radiation-induced intestinal injury. Immunohistochemical staining showed that M1 improved cell proliferation, reduced cell apoptosis, and enhanced the epithelial integrity of intestinal crypts. This study provides a promising oral ROS-sensitive agent for acute intestinal radiation syndrome.

## 1. Introduction

The widespread use of nuclear energy in aerospace, military, medical, and other fields leads to the increasing exposure to human beings from ionizing radiation (IR) [1,2,3,4,5]. Meanwhile, as a dominant cancer treatment, radiotherapy can directly generate large amounts of ROS, contributing to DNA double-strand breakage, intracellular oxidative stress, and cells apoptosis [6,7]. The side-effects of radiotherapy, especially presenting as hematopoietic system suppression and gastrointestinal dysfunction, can be lethal to patients [8,9]. Therefore, the development of radiation protective medicine has become a crucial medical goal.

Much progress has been made in the synthesis and screening of radioprotective compounds, such as aminothiols [10,11,12,13], polyphenols [14,15], and growth factors [16,17]. As radical scavengers, aminothiols provide H atoms to scour hydroxyl radicals or other ROS [18,19,20]. Amifostine is a radioprotective agent approved by the U.S. Food and Drug Administration (FDA) for use as a radiation protector [21,22]. Amifostine is an organic thiophosphate prodrug that is dephosphorized by plasma membrane alkaline phosphatase to the free thiol metabolite (WR-1065) [23,24]. Amifostine is unlikely to be fully realized as a radioprotective agent since its administration is mainly intravenous. The maximum radioprotective effect of amifostine is observed when it is administered by a 15-min infusion starting 30 min to 60 min before irradiation [25]. Not only is amifostine rapidly cleared from the body, but it also has a short distribution half-life of 0.9 min when administered as a high dose or 15 min intravenous (i.v.) infusion. Regardless of the convenient and practical route for orally administration, side effects of intravenous administration, such as vomiting, nausea, and hypotension, are also significantly observed upon such administration [26]. The oral delivery of amifostine has been widely researched, such as in the form of enteric-soluble amifostine capsules, which protect amifostine from acids and enzymes in the stomach and are released in the intestine [27,28]. Oral delivery of radioprotective compounds can be achieved through the thioester bond hydrolysis reaction. The hydrolysis reaction of compounds in the intestinal tract releases active molecules, leading to free thiol to effectively scavenge ROS.

**Figure 1:**
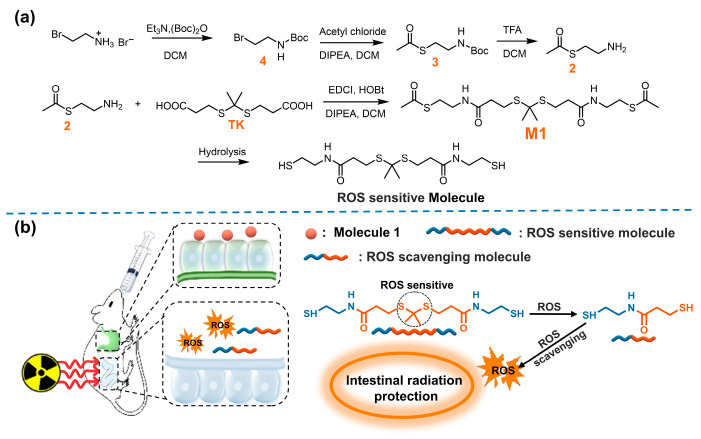
(**a**) The synthesis of M1; (**b**) schematic illustration of ROS scavenging by M1 in vivo.

Thioketal (TK) is a thiol-based ROS-responsive bond, which has been widely reported in drug delivery and therapy. Nevertheless, molecules containing both sulfhydryl and TK as an antioxidant have not been reported [29,30,31]. In this work, TK was introduced into thiol active molecules to obtain ROS-sensitive compounds [32,33,34,35]. In the case of ROS eruptions caused by irradiation exposure, a cascade reaction can be activated to cause the rupture of the active molecule, and the resulting aminothiol molecule can further remove ROS (Figure 1).

In this study, we designed, synthesized, and evaluated a new oral ROS-responsive radioprotective compound. As shown in Figure 1a, compound M1 was obtained through the reaction of intermediate compound 2 and TK and hydrolyzed under alkaline conditions to obtain ROS-responsive active molecules. The compound exists stably in acidic environments and can be hydrolyzed to release active molecules with abundant free thiol in intestinal alkaline environments. In the case of ROS eruption after irradiation, the TK response group breaks and forms active molecules capable of scavenging ROS (Figure 1b). Therefore, the ROS-responsive molecule was expected to be a promising candidate radioprotector by oral administration.

## 2. Materials and Methods

### 2.1. Materials

The 3-Bromopropylamine hydrobromide, N-(3-dimethylaminopropyl)-N′-ethylcarbodiimide hydrochloride (EDCI), 1-hydroxybenzotriazole monohydrate (HOBt), N,N-Diisopropylethylamine (DIPEA), 5,5′-dithiobis-2-nitrobenoic acid (DTNB), potassium thioacetate, 3-Mercaptopropionic acid, and propanone were purchased from Shanghai Aladdin with purity of 99%. Reagents were purchased from Tianjin Jiangtian Chemical Technology Co. Ltd without further purification, unless otherwise noted. Silica gel (300–400 mesh) was used for column chromatography. The HIEC-6 cell line was provided by the Institute of Basic Medical Sciences, Chinese Academy of Medical Sciences (Beijing, China). Fetal bovine serum (FBS), 3-Dulbecco’s Modified Eagle’s Medium (DMEM) and trypsin were obtained from Invitrogen Corporation. The 4′, 6-diamidino-2-phenylindole (DAPI), neutral red (NR), and 2′, 7′-dichlorofluorescin diacetate (DCFH-DA) were purchased from Sigma-Aldrich Co., Ltd. (St Louis, MO, USA). Male C57BL/6 mice (6–8 weeks) were purchased from Beijing Vital River Laboratory Animal Technology (Beijing, China) and acclimated to the environment (specific-pathogen-free, 22 °C ± 2 °C, 50% humidity, and 12 h light/dark cycle) for seven days with free access to a standard diet. The animal ethics number is IRM-DWLL-2022039.

### 2.2. Characterization

The ^1^H NMR and ^13^C NMR spectra were recorded on a Varian Inova 500 MHz NMR spectrometer using tetramethylsilane (TMS) as the internal standard. Multiplicities for proton signals are abbreviated as s, d, t, q, p, and m for singlet, doublet, triplet, quartet, pentet, and multiplet, respectively. UV/vis absorption spectra were recorded on an Agilent Technologies Cary 500 UV/vis spectrophotometer. The Infinite F200 multimode plate reader was used to measure the cell viability. Images of cells were collected using a confocal microscope under a 63 × oil microscope (Nikon, Eclipse Ti2, Tokyo, Japan).

### 2.3. Synthesis and Characterization of M1

Synthesis of compound 4: The synthesis of compound 4 has been reported previously in the literature [36]. The 2-aminoethane-1-thiol hydrochloride (5.00 g, 44.01 mmol) and sodium bicarbonate (7.39 g, 88.03 mmol) were mixed in THF/H_2_O (*v*/*v* = 1/1, 100 mL) with Et_3_N (11.53 g, 52.82 mmol). The mixture was stirred at room temperature for 12 h. The mixed system was washed by adding pure water and dried by anhydrous sodium sulfate for the organic phase. The product appears as a white solid.

Synthesis of compound 3: Compound 4 (5.0 g, 28.21 mmol) and potassium thioacetate (3.54 g, 31.03 mmol) were mixed in 10 mL DCM with excess DIPEA at room temperature for 12 h. After the reaction was complete, an equal volume of water was added to the reaction solution, and the organic phase was dried and suspended to yield compound 4 as a reddish-brown oil. The ^1^H NMR spectrum and ^13^C NMR spectrum of compound 3 in DMSO are shown in Figures S1 and S2, respectively. ^1^H NMR (300 MHz, DMSO) δ 3.33 (s, 1H), 3.05 (dd, J = 13.0, 6.3 Hz, 2H), 2.87 (t, J = 6.8 Hz, 2H), 2.32 (s, 3H), 1.37 (s, 9H). ^13^C NMR (75 MHz, DMSO) δ 195.62, 155.91, 138.18, 78.34, 31.00, 29.07, 28.67.

Synthesis of compound 2: Compound 3 was dissolved in 10 mL DCM and an equal volume of TFA was added. The mixture was stirred at room temperature for 2 h, and the product was concentrated under reduced pressure to obtain a brown oil. The ^1^H NMR spectrum and ^13^C NMR spectrum of compound 2 in DMSO are shown in Figures S3 and S4, respectively. ^1^H NMR (300 MHz, DMSO) δ 8.63–8.07 (m, 3H), 3.11 (t, J = 7.2 Hz, 2H), 2.92 (s, 2H), 2.37 (s, 3H). ^13^C NMR (75 MHz, DMSO) δ 195.31, 38.68, 31.03, 26.15.

Synthesis of compound TK: The 3-Mercaptopropionic acid (4.90 g, 46.17 mmol) was mixed in propanone (5.36 g, 92.33 mmol) with dry hydrogen chloride, and the mixture was stirred at room temperature for 6 h. After the reaction, the mixture was filtered to obtain a white solid. The ^1^H NMR spectrum and ^13^C NMR spectrum of TK in DMSO are shown in Figures S5 and S6, respectively. ^1^H NMR (300 MHz, DMSO) δ 7.27 (s, 2H), 2.92 (t, J = 7.4 Hz, 4H), 2.69 (t, J = 7.4 Hz, 4H), 1.61 (s, 6H). ^13^C NMR (75 MHz, DMSO) δ 173.52, 56.19, 34.23, 30.92, 25.49.

Synthesis of compound M1: TK (500 mg, 1.98 mmol) was suspended in DCM (30 mL), and compound 2 (519.53 mg, 4.36 mmol), EDCI (835.66 mg, 4.36 mmol), HOBt (589.03 mg, 4.36 mmol), and DIPEA (563.41 mg, 4.36 mmol) were added to it. The mixture was stirred vigorously at room temperature for 24 h under N_2_ atmosphere, after which the solvent was removed by vacuum rotary evaporation. The crude material was purified by column chromatography using MeOH/CH_2_Cl_2_ (1:20) as an eluent to yield M1 as a white solid. The ^1^H NMR spectrum and ^13^C NMR spectrum of M1 in DMSO are shown in Figures S7 and S8, respectively. ^1^H NMR (300 MHz, DMSO) δ 8.13 (t, J = 5.6 Hz, 2H), 3.18 (dd, J = 12.7, 6.7 Hz, 4H), 2.90 (t, J = 6.9 Hz, 4H), 2.73 (t, J = 7.4 Hz, 4H), 2.37–2.27 (m, 10H), 1.52 (s, 6H). ^13^C NMR (75 MHz, DMSO) δ 195.56, 171.00, 56.05, 38.71, 35.66, 31.09, 31.03, 28.69, 26.00.

### 2.4. Cell Culture and Cytotoxicity Evaluation

HIEC-6 cells were cultured in Dulbecco’s Modified Eagle Medium (DMEM) supplemented with 1% penicillin-streptomycin and 10% (*v*:*v*) fetal bovine serum in an incubator at a temperature of 37 °C with 5% CO_2_. To test cytotoxicity of the M1, HIEC-6 cells were seeded on 96-well plates and then were incubated with M1 by various concentrations of 0, 0.01, 0.05, 0.1, 0.5, 1.0, 5.0, and 10 mM for 24, 36, and 72 h at 37 °C under 5% CO_2_. Subsequently, the NR solution (20 μL) was added to the cells incubated for 2.5 h, and then washed with PBS. The cells were lysated by adding to 150 μL lysis buffer at room temperature and were detected at 540 nm with a microplate reader (TECAN, Infinite 200 Pro, Männedorf, Switzerland).

### 2.5. Irradiation Conditions

Cells were irradiated with γ-ray irradiator (0.99 Gy/min, Canada Gammacell-40) at various doses (2, 4, 6, 8, and 10 Gy), and the animals were exposed to a single abdominal irradiation (ABI) of 13 Gy.

### 2.6. Sulfhydryl Group Detection

DTNB was chosen as the detection reagent of the free sulfhydryl groups, and glutathione was used as the reference molecule. The sulfhydryl group can react with DTNB to form yellow 2-nitro-5-sulfhydryl benzoic acid with a characteristic absorption peak at 412 nm, and the concentration of the total sulfhydryl group can be quantitatively analyzed through the change of absorbance value. DTNB (0.198 g) was accurately weighed and prepared into 50 mL solution with 50 mM Na_2_HPO_4_ (pH = 7.0). The solution was stored in a brown bottle and stored in the dark at low temperature. The various concentrations (0.01, 0.05, 0.1, 0.5, 1, 5, and 10 mM) of glutathione were reacted with DTNB, and the absorbance at 412 nm was measured to produce a standard curve.

### 2.7. ROS Detection

The HIEC-6 cells were planted in 6-well plates at a density of 2.0 × 10^5^ cells/well for 24 h. After attachment, the cells were incubated with the M1 PBS solution at different concentrations of 0.1 and 0.5 mM prior to irradiation (6 and 8 Gy) for 24 h. Then, the cells were washed with PBS three times, and 1 mL of 5 µM DCFH-DA was added at 37 °C for 20 min. After incubation, the cells were washed by PBS three times, and the fluorescence intensity was measured at 490 nm with an Infinite F200 multimode plate reader.

### 2.8. Colony Formation Assay

The HIEC-6 cells were planted in a 6-well plate with a density of 500 cells/well for 24 h. After attachment, the cells were incubated with the solution of M1 at different concentrations of 0.1 and 0.5 mM prior to irradiation (0, 4, 6, 8, and 10 Gy). Ten days after irradiation, the cells were fixed with 1 mL paraformaldehyde (4%) for 60 min and stained with crystal violet (0.5%) for 10 min, and then, the cells were washed with distilled water twice after discarding the dye solution. The number of colonies was calculated by Image Pro Plus 8.0 software (Media Cybernetics, Maryland, USA).

### 2.9. Apoptosis Assays

Apoptosis assay was performed according to the instructions of the Annexin V-FITC Apoptosis Detection Kit (Solarbio, CA1020, Beijing, China). Briefly, HIEC-6 cells were seeded in 6-well plates at a density of 15 × 10^4^ cells/well. After attachment, the cells were co-incubated with M1 (0.1 mM and 0.5 mM) for 30 min before irradiation and were incubated for 24 h after irradiation. Then, the cells were collected to stain FITC and PI. The data acquisitions were analyzed through FlowJo VX software (Becton-Dickinsom, New Jersey, USA).

### 2.10. Alkaline Comet Assay

Single-cell gel electrophoresis assay was used to investigate damaged DNA according to the manufacturer’s instructions. Briefly, HIEC-6 cells were seeded in 6-well plates at a density of 15 × 10^4^ cells/well. After attachment, the cells were co-incubated with M1 for 30 min before irradiation. After irradiation, the cells were collected and then suspended to PBS. An amount of 100 μL agarose gel with normal melting point was spread on CometSlide™ slides and was set at 4 °C for 10 min. The 10 μL cell suspension and 75 μL low melting point agarose were mixed evenly and dropped on the slides. The slides were set at 4 °C for solidification for 10 min; then, the 75 μL low melting point agarose was dropped onto the slides, covered with the cover glass, and solidified for 20 min at 4 °C. The slides were soaked in a pre-cooled lysis buffer for 2 h at 4 °C and subsequently placed in a freshly prepared alkaline lysis solution (1 mmol/L EDTA, 300 mmol/L NaOH, in H_2_O) for 60 min. The slides were stored in alkaline electrophoresis solution, and electrophoresis was conducted at 30 V for 30 min. After electrophoresis, the slides were neutralized in 0.4 mM Tris-HCl buffer (pH = 7.5). Then, the 20 µL PI solution was dropped on the slides for dark staining for 10 min. Finally, the slides were examined through fluorescence microscope, and DNA damage was assessed using the Comet Assay Software Project (CASP 1.2.3, Trevigen, Maryland, USA).

### 2.11. Detection of γ-H2AX

Histone H2AX (γ-H2AX) was used to investigate DNA double strand breaks (DSBs). The HIEC-6 cells were incubated with a density of 10 × 10^5^ cells per confocal microscope dish. After attachment, the cells were co-incubated with M1 for 30 min before irradiation. Then, 1 h after irradiation, the cells were fixed with 4% paraformaldehyde for 20 min and then were washed with PBS and treated with 0.2% Triton-X 100 for 15 min. After PBS washing, the cells incubated with rabbit polyclonal γ-H2AX (phospho S139) primary antibody (dilution 1: 1000; cat. No. ab2893; Abcam, Cambridge, MA, USA) at 4 °C overnight. After discarding the primary antibody, the cells were gently washed with PBS three times and were incubated with goat anti-rabbit secondary antibody (diluted 1:2000; cat. No. ab6939; Abcam) at room temperature for 1 h. The nuclei were counterstained with DAPI (cat. No. C0065, Solarbio, Beijing, China). Images of the cells were collected using a confocal microscope under a 63 × oil microscope (Nikon, Eclipse Ti2, Tokyo, Japan). The foci in each picture were analyzed by Image Pro Plus 8.0 software (Media Cybernetics, Maryland, USA).

### 2.12. Mice

Mice were administrated with 0.2 mL 125, 250, 500 mg/kg M1 solution by oral gavage or were intraperitoneally injected with 250 mg/kg amifostine (positive control, named as Ami) 30 min before irradiation. In the control group, the blank solvent was a mixture of 10% DMSO, 40% PEG300, 5% Tween-80, and 45% saline. Mice were anesthetized and abdominally irradiated with a single dose of 13 Gy γ-ray by a Gammacell 40 Exactor (Atomic Energy of Canada, Chalk River, Ottawa, ON, Canada) at a rate of 0.99 Gy/min. All animal experiments were authorized by the local animal care and use committee.

### 2.13. Histology

The small intestines of the mice were collected after three days of 13 Gy ABI and hematoxylin and eosin (H&E) staining was performed on the small intestinal sections. Morphological analysis of the small intestines (including villus length and crypt) was performed using ImageJ 1.37 software (NIH, Bethesda, USA).

### 2.14. Immunohistochemical Analysis

The duodenums of the mice were embedded in paraffin and incubated with anti-Ki67 (1:300 dilution; Novus, Littleton, CO, USA). Positive cells were detected by DAB kit (Sigma-Aldrich, St. Louis, MO, USA). The TUNEL apoptosis detection kit was used for TUNEL staining, and the stained small intestines were observed under an optical microscope.

### 2.15. Statistical Analysis

All statistical analyses were performed using GraphPad Prism 8.0 (GraphPad Software, San Diego, CA, USA). Values were expressed as means ± SD. T-test was used for comparisons between two groups, and multiple-group comparisons were performed by one-way ANOVA. Statistically significant differences were considered when *p* value < 0.05. Significance thresholds of * *p* < 0.05, ** *p* < 0.01, *** *p* < 0.001, and **** *p* < 0.001 were applied.

## 3. Results

### 3.1. Solution Characterization of M1 on pH-Sensitive and ROS-Responsive

**Figure 2:**
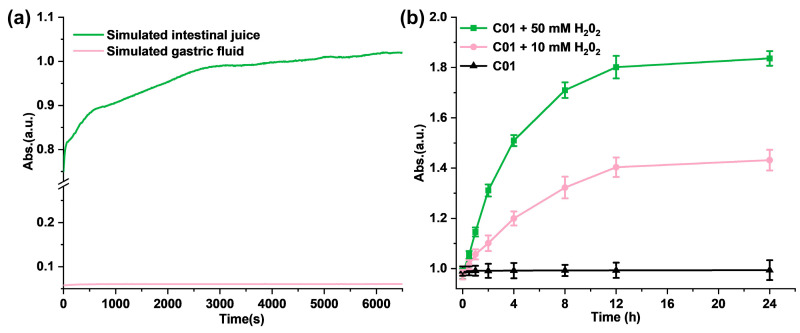
(**a**) The hydrolysis kinetics of M1 in solution in simulated gastric juice and simulated intestinal fluid; (**b**) ROS-responsive fragmentation of M1 molecules in PBS in vitro.

Firstly, we investigated the pH sensitivity of M1 in solution. To verify the releasing process of the thiol group from the synthesized M1, glutathione was selected as the reference molecule, and DTNB was chosen as the detection reagent to produce a standard curve. As shown in Figure S9, the absorbance at 412 nm was proportional to the concentration of glutathione. Next, we studied the stability of M1 under a simulative stomach condition (pH = 1.2). There was no difference observed in the ^1^HNMR spectra of M1 after the hydrochloric acid was added, which conformed with the stability of M1 in the simulative stomach condition (Figure S10). In addition, we evaluated the hydrolysis kinetics of M1 in various pH solutions. As shown in Figure 2a, the hydrolysis of M1 (2.0 mM) in simulated gastric juice was hardly observed within 100 min, while, in the simulated intestinal fluid (pH = 8.4), the absorbance value at 412 nm gradually increased in a time-dependent manner and reached plateau after 3000 s. According to the standard curve in Figure S9, the concentration of the sulfhydryl group is 1.75 mM at 100 min. The above results provide a demonstration that M1 is pH-sensitive as it is stable in the simulated gastric fluid but not in the simulated intestinal juice.

Meanwhile, to evaluate the ROS-response process in vitro, H_2_O_2_ solutions were added to M1 PBS solution with a concentration of 5.0 mM to trigger the release of the free thiol and further quantify the free sulfhydryl group with DTNB. As shown in Figure 2b, the absorbance in 412 nm increased significantly with the H_2_O_2_ addition compared with the control solution. In addition, the higher concentration of H_2_O_2_ (50 mM) induced more free thiols of M1; the absorbance of the reaction product at 412 nm is positively correlated with the time and the concentration of H_2_O_2_. It follows that the addition of H_2_O_2_ promotes the disintegration of M1 molecules and releases the free sulfhydryl groups.

### 3.2. M1 Inhibits the Reduction in Clone Formation by Scavenging ROS

Firstly, the cytotoxicity of M1 was measured on HIEC-6 cells by NR assay at various concentrations. As shown in Figure S11, no obvious cytotoxicity was observed less than 0.1 mM of M1 for 24, 48, and 72 h treatment. The IC_50_ of M1 was calculated as 5.8 mM, and non-toxic doses of M1 (0.1 and 0.5mM) were used in the subsequent assays.

**Figure 3:**
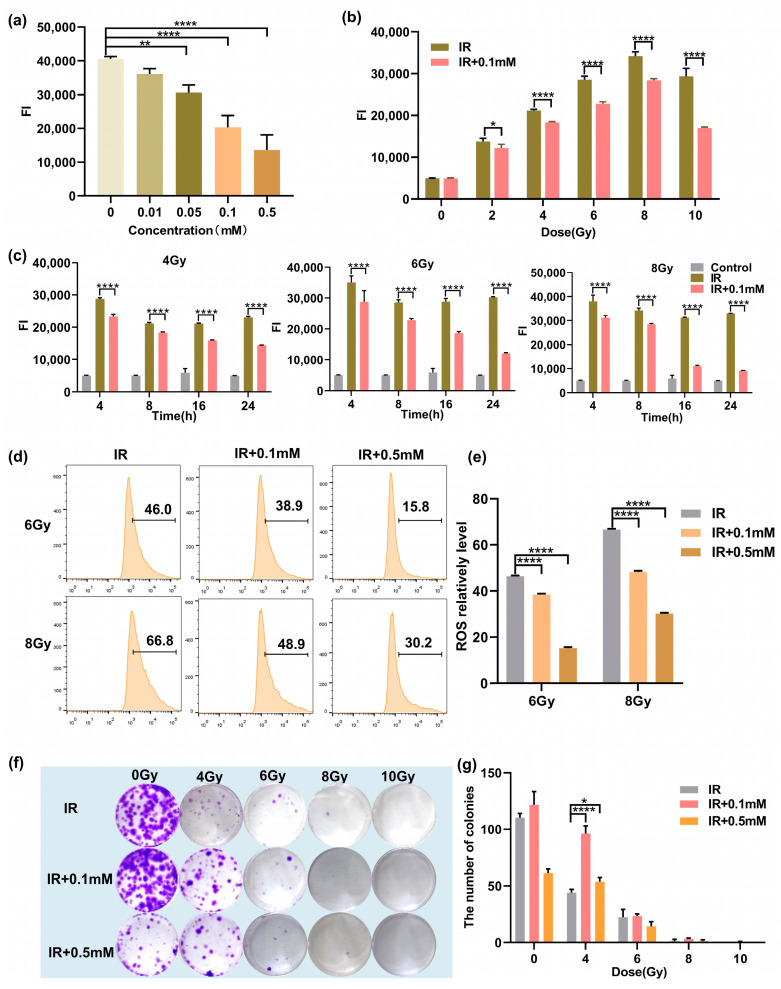
(**a**) The ROS levels in cells treated with various concentrations of M1 at 8 h after 4 Gy irradiation. (**b**) The ROS levels in M1 treated and untreated cells at 8 h after various doses of irradiation. (**c**) The ROS levels in M1 treated and untreated cells at various times after 4, 6, and 8 Gy irradiation. (**d**) Flow cytometry ROS analysis of HIEC-6 cells treated with groups of IR, IR + 0.1 mM, and IR + 0.5 mM at 24 h after 6 and 8 Gy irradiation (numbers represent ROS level). (**e**) Quantitative analysis of ROS level from (**d**). (**f**) Typical clone images of HIEC-6 cells belong to groups of IR, IR + 0.1 mM, and IR + 0.5 mM at 10 days after 0, 4, 6, 8, and 10 Gy irradiation. (**g**) Quantitative analysis of survival fractions from (**f**). Results are presented as the mean ± SD in triplicate and analyzed by one-way ANOVA.

Next, we studied the ROS levels of the cells after irradiation. First, the ROS levels in cells treated with various concentrations of 0, 0.01, 0.05, 0.1, and 0.5 mM at 8 h after 4 Gy irradiation were measured. As shown in Figure 3a, it was observed that 0.05 mM M1 could significantly reduce the ROS level induced by radiation. With the increased M1, the ROS level gradually decreased in a concentration-dependent manner. ROS induced by 4 Gy radiation was cleared about 50% by treatment with 0.5 mM M1. Then, we studied the ROS-scavenging ability of M1 at different irradiation doses. As shown in Figure 3b, 8 h after irradiation, compared with the IR group, the ROS level could be reduced in the administration (IR + 0.1 mM) group at the M1 concentration of 0.1 mM. We further investigated the changes in the ROS levels at different time points. As shown in Figure 3c, the ROS level was gradiently induced by various radiation doses, and a peak level of ROS was detected at 4 h after radiation. With the prolonged time after radiation, the ROS level in the administration group (IR + 0.1mM) was significantly lower than that in the irradiation group (IR), which proved that ROS-responsive M1 showed the ability to continuously clear ROS.

Meanwhile, ROS levels in the IR group, the IR + 0.1 mM group, and the IR + 0.5 mM group were quantitatively analyzed by flow cytometry. As shown in Figure 3d, compared with the IR group, the ROS levels in the IR + 0.1 mM group and the IR + 0.5 mM group were significantly reduced, and the increased drug concentration could promote the clearance of ROS. Quantitative analysis of ROS level is shown in Figure 3e, and results are presented as the mean ± SD in triplicate.

The colony-forming cell assay was used to evaluate the proliferation and differentiation pattern of irradiated HIEC-6 cells exposed with/without M1 treatment. As shown in Figure 3f,g, HIEC-6 cells in the IR + 0.1 mM group irradiated with 0, 4, 6, 8, and 10 Gy had significantly higher survival fraction than the irradiated group. For the IR + 0.5 mM group, 0.5 mM M1 presented a cytotoxic effect compared with the control group (0 Gy), while higher colony counts were observed compared to the group exposed with 4Gy radiation. It proved that 0.1mM M1 could significantly rescue cell proliferation suppression induced by 4 Gy radiation without cytotoxicity.

### 3.3. M1 Ameliorates Apoptosis Induced by Irradiation

**Figure 4:**
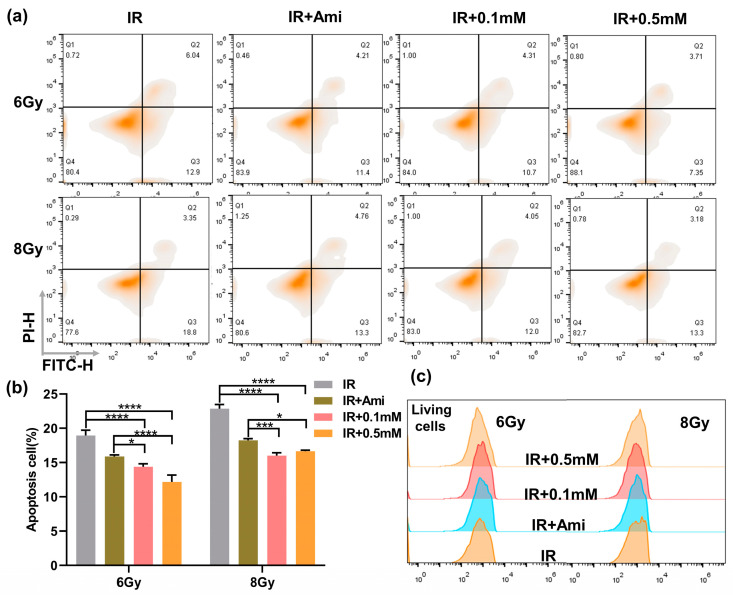
(**a**) Flow cytometry apoptosis analysis of HIEC-6 cells treated with groups of IR, IR + Ami, IR + 0.1 mM, and IR + 0.5 mM at 24 h after 6 and 8 Gy irradiation. (**b**) Living cells analysis from (**a**). (**c**) Quantitative analysis of apoptosis cells from Figure 4a. Results are presented as the mean ± SD in triplicate.

It has been reported that excessive ROS can attack mitochondria and damage mitochondrial function, leading to cell death, while the thiol can remove ROS to prevent the damage to mitochondria [37]. In this work, the flow cytometry was used to investigate whether M1 could inhibit radiation-induced apoptosis; HIEC-6 cells were counterstained with FITC/PI, and amifostine (Ami) was used as a positive control. As shown in Figure 4a,b, compared with the IR group, under 6 Gy irradiation, the percentage of apoptotic HIEC-6 cells treated with 0.1 or 0.5 mM M1 solution decreased from 18.95% to 14.38% and 12.17%, respectively, and decreased from 22.86% to 16.0% and 16.65%, respectively, under 8 Gy irradiation. The 0.5 mM M1 presented a lower apoptosis rate compared with amifostine as the positive control.

At the same time, the proportion of living cells were statistically analyzed; as shown in Figure 4c, the proportion of living cells in the IR + Ami, IR + 0.1 mM, and IR + 0.5 mM groups were higher than in the IR group, which provided evidence for M1 to protect normal cells by removing ROS and thus exert a radiation protection effect.

### 3.4. M1 Attenuates Radiation-Induced DNA Damage

**Figure 5:**
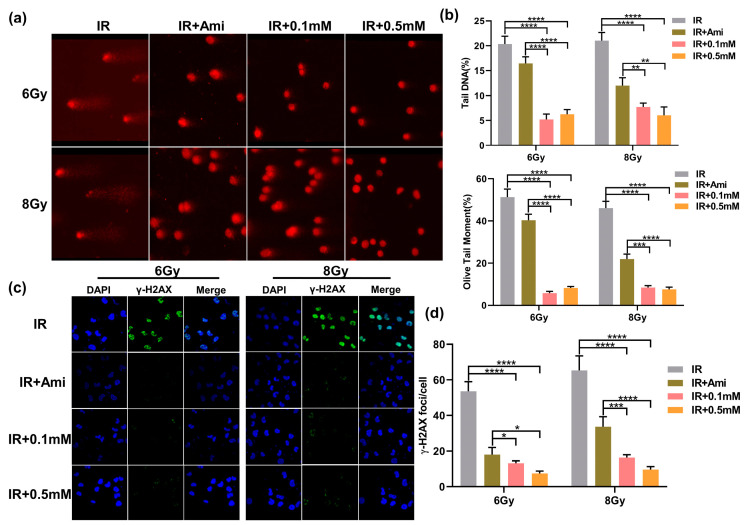
(**a**) Typical comet assay images of HIEC-6 cells treated with groups of IR, IR + Ami, IR + 0.1 mM, and IR + 0.5 mM after 6 and 8 Gy irradiation. (**b**) Quantitative analysis of olive tail moment and tail DNA from (**a**). Results are expressed as the mean ± SD of 50 replicates. (**c**) CLSM microscopy observation of γ-H2AX immunofluorescence of HIEC-6 cells treated with groups of IR, IR + Ami, IR + 0.1 mM, and IR + 0.5 mM after 6 and 8 Gy irradiation. (**d**) Quantitative analysis of γ-H2AX foci from Figure 5c. Results are expressed as the mean ± SD of 20 replicates.

Irradiation can directly lead to DNA DSBs, and the generation of explosive ROS may cause indirect damage to DNA. The damaged DNA can be uncoiled in the unwinding solution and then migrated faster to form small tails under the action of the external electric field. Therefore, this phenomenon could be used to visually judge the extent of DNA damage. Since the shape of the cells after electrophoresis is similar to that of a comet, this experiment was called a comet assay [38]. In this work, comet assay was used to evaluate the DNA damage at the single cell level, which is commonly represented by two indexes: Olive tail moment and tail DNA content. As shown in Figure 5a, the HIEC-6 cells were severely damaged after irradiation, and the Olive tail moment and tail DNA were remarkably higher compared with cells pretreated with M1 or amifostine. In comparison, 30 min before irradiation, co-incubation of 0.1 and 0.5 mM M1 solutions with cells could significantly reduce tail DNA content, shorten Olive tail distance, and obviously alleviate DNA damage caused by radiation (Figure 5b).

Histone γ-H2AX was involved in the repair of irradiation-induced DNA DSBs damage, which was an early marker of DSBs. To evaluate the radioprotective effect of M1, γ-H2AX rabbit monoclonal antibody labeled with green fluorescence was applied to examine DNA DSBs. It is reported that the initial peak of the γ-H2AX foci mainly appeared at 0.5 h and can be observed at 24 h after irradiation in mammalian cells [39]. In this experiment, the γ-H2AX foci labeled by green fluorescence in the cell nuclei of each group were detected after 1h irradiation. As shown in Figure 5c, the DAPI signals and γ-H2AX signals were represented by blue dots and green spots, respectively, and the merge signals were represented by γ-H2AX signals on the DAPI background. Compared with the IR group, the radiation-induced γ-H2AX foci fluorescence intensity was significantly reduced in cells pretreated with 0.1 and 0.5 mM of M1 or 0.1 mM amifostine. In Figure 5d, under 6 Gy irradiation, the number of γ-H2AX foci was reduced from 53.49 to 13.27, 7.35, and 18.03, respectively, in cells pretreated with 0.1 and 0.5 mM of M1 and 0.1 mM amifostine. While under 8 Gy irradiation, this value decreased from 65.28 to 16.38, 9.68, and 33.79, respectively. The statistical analysis of the γ-H2AX foci provided evidence that M1 could effectively reduce DNA DSBs damage caused by irradiation.

### 3.5. M1 Ameliorates Intestine Structural Injuries Induced by 13 Gy ABI

**Figure 6:**
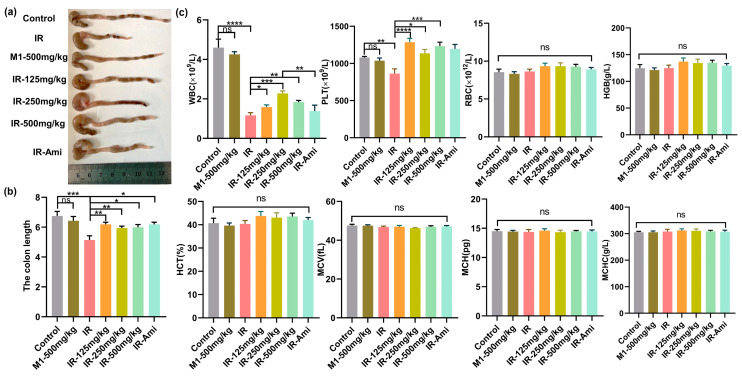
(**a**–**c**) Colon tissues, lengths, and hematopoietic system analyses of mice in control, IR, M1-500 mg/kg, IR + 125 mg/kg, IR + 250 mg/kg, IR + 500 mg/kg, and IR + Ami groups three days after 13 Gy ABI, *n* = 6 per group.

The gastrointestinal system has rapidly proliferating and differentiating cells, which make it one of the most vulnerable organs in the body. Irradiation disrupts the GI system by damaging proliferating stem cells of the crypts that alter the histology and physiology of the intestine. The length of the colon is utilized to evaluate the pathological changes of the intestinal structure. Intestinal inflammation caused by local irradiation can lead to colitis presenting as body weight loss, colon length shortening, diarrhea, and bloody stools. To explore the gut protective effects of M1 in vivo, we evaluated the colon lengths in irradiated male mice treated with and without M1 administration. The colonic lengths of C57BL/6 mice were measured three days after 13 Gy ABI. Colon images for all groups are shown in Figure S12. Compared with the control group, the colon lengths were significantly shortened in the IR group from 6.75 cm to 5.13 cm. As shown in Figure 6a,b, irradiation-induced colonic shortening was significantly reversed from the starting dose of 125 mg/kg M1.

In addition, the hematopoietic system is sensitive to ionizing radiation. Therefore, we studied the effect of irradiation in mice treated with and without M1 on the hematopoietic system by extracting blood from the retro-orbital veins three days after irradiation. Mice were treated with amifostine as the positive control and various doses of M1 30 min prior to irradiation. As shown in Figure 6c, compared with the control group, WBC counts as the early marker during radiation exposure were obviously reduced in irradiated mice, while M1 pretreatment significantly rescued WBC reduction with 250 mg/kg. After oral administration, M1 was relatively stable in gastric juice and was irreversibly hydrolyzed in the alkaline environment of the intestinal system, releasing free sulfhydryl groups for ROS removal. Additionally, the breakage of the ROS-sensitive thioacetone bond in the M1 was caused by bursting ROS induced by radiation, leading to extra release of free thiol groups to scavenge ROS. Therefore, M1 is verified in vivo as a pH-sensitive and ROS-responded dual-function prodrug of ROS scavenger.

### 3.6. M1 Improves Cell Proliferation, Reduces Cell Apoptosis, and Enhances Epithelial Integrity in Intestinal Crypts after 13 Gy ABI

**Figure 7:**
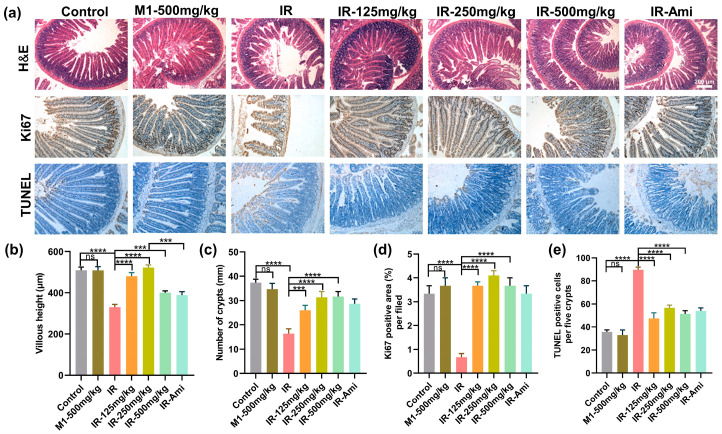
(**a**) Representative images showing the structure in cross-sections of the small intestine with hematoxylin and eosin (H&E), Ki67, and TUNEL staining. Scale bar: 200 μm. (**b**–**e**) Histogram showing the number of crypts, villus lengths, and Ki67 positive and TUNEL positive cells in intestinal sections from the control, IR, M1-500 mg/kg, IR + 125 mg/kg, IR + 250 mg/kg, IR + 500 mg/kg, and IR + Ami groups. The results are represented as mean ± SEM, *n* = 6 mice per group. *** *p* < 0.001, **** *p* < 0.0001 by one-way analysis of variance (ANOVA).

We further investigate histological changes within the small intestines of mice from different treatment groups exposed with 13 Gy ABI. At three-days post-irradiation, H&E staining was performed on the small intestine of different treatment groups. As shown in Figure 7a, compared with the control group, pathological phenomena, such as villus peeling and crypt atrophy, were observed in the small intestines of the IR group, while the villus structures of the small intestines of the M1 administration groups were relatively intact. In addition, the villus lengths and crypt numbers were also statistically analyzed, as shown in Figure 7b,c; compared with the IR group, the damage to the villi and crypts in the irradiation group treated with MI was significantly weakened.

Immunohistochemical localization of the Ki67 antibody was used to detect the small intestine sections, and Ki67 expression stained by IHC was utilized to evaluate the proliferation and apoptosis of crypt cells at three days after 13 Gy ABI. As shown in Figure 7a,d, compared with the control group, the Ki67 positive cells in the crypts of the IR group were significantly decreased, while various doses of M1 pretreatment rescued the Ki67+ cells counts reduction remarkably. Similarly, the results of TUNEL staining of the small intestine (Figure 7e) demonstrated that M1 effectively prevented irradiation-induced enterocytes apoptosis. All these results could prove that M1 can alleviate villus rupture and crypt injury in the small intestine caused by high-dose local irradiation.

## 4. Conclusions

In total, we have developed a novel pH-sensitive and ROS-responded prodrug M1 as an oral radioprotective candidate for radiation-induced enteritis. Results in vitro shown that M1 was relatively stable 100 min in the acidic environment and could be hydrolyzed rapidly in the intestinal environment to form active molecules rich in thiols. In addition, the TK bond of M1 could also be further triggered by ROS induced by H_2_O_2_ solution effectively, contributing to extra ROS-scavenging ability. Due to excellent biocompatibility and ROS-scavenging ability, M1 can reduce the level of ROS produced by local radiation and prevent radiation-induced acute enteritis in mice. Results showed that M1 significantly attenuated irradiation-induced acute intestinal injury (13 Gy ABI) even at lower doses (125 mg/kg), and hemopoietic system suppression also benefited from the M1 pretreatment by rescuing the WBC counts reduction. Therefore, our study provides a promising candidate for effective radiation protection in the oral treatment of radiation enteritis.

## Data Availability

The data supporting the conclusions of this paper are provided in this paper and its additional files.

## Supplementary Materials

The following supporting information can be downloaded at: https://www.mdpi.com/article/10.3390/antiox11112145/s1, Figure S1: ^1^H NMR spectrum of compound 3 in DMSO; Figure S2: ^13^C NMR spectrum of compound 3 in DMSO; Figure S3: ^1^H NMR spectrum of compound 2 in DMSO; Figure S4: ^13^C NMR spectrum of compound 2 in DMSO; Figure S5: ^1^H NMR spectrum of TK in DMSO; Figure S6: ^13^C NMR spectrum of TK in DMSO; Figure S7: ^1^H NMR spectrum of M1 in DMSO; Figure S8: ^13^C NMR spectrum of M1 in DMSO; Figure S9: Standard curve between concentrations of glutathione and absorbance at 412 nm; Figure S10: ^1^H NMR spectrum of M1 with HCl in DMSO; Figure S11: Cytotoxicity assays of M1 with HIEC-6 cells; Figure S12: Colon tissues of mice in Control, IR, M1-500 mg/kg, IR + 125 mg/kg, IR + 250 mg/kg, IR + 500 mg/kg and IR + Ami groups three days after 13 Gy ABI, *n* = 6 per group.
