# Supplementary material for: ROS-Responsive and pH-Sensitive Aminothiols Dual-Prodrug for Radiation Enteritis
Source: Antioxidants (Basel). 2022 Oct 29;11(11):2145. doi: 10.3390/antiox11112145 (PMC9686648; doi:10.3390/antiox11112145)
Supplement: Supplementary file 1 [file antioxidants-11-02145-s001.zip › antioxidants-1965924-supplementary.pdf]

ROS-responsive and pH-sensitive aminothiols dual-prodrug for radiation enteritis

Yuanfang Chen, Yuwei Yang, Haikang Tang, Ziqi Zhang, Xiaoliang Zhou*, Wenqing Xu*

1. Institute of Radiation Medicine, Chinese Academy of Medical Sciences and Peking Union Medical College, Tianjin 300192, China

* Correspondence: zhouxiaoliang@irm-cams.ac.cn (X.Z.); xuwenqing@irm-cams.ac.cn (W.X.).

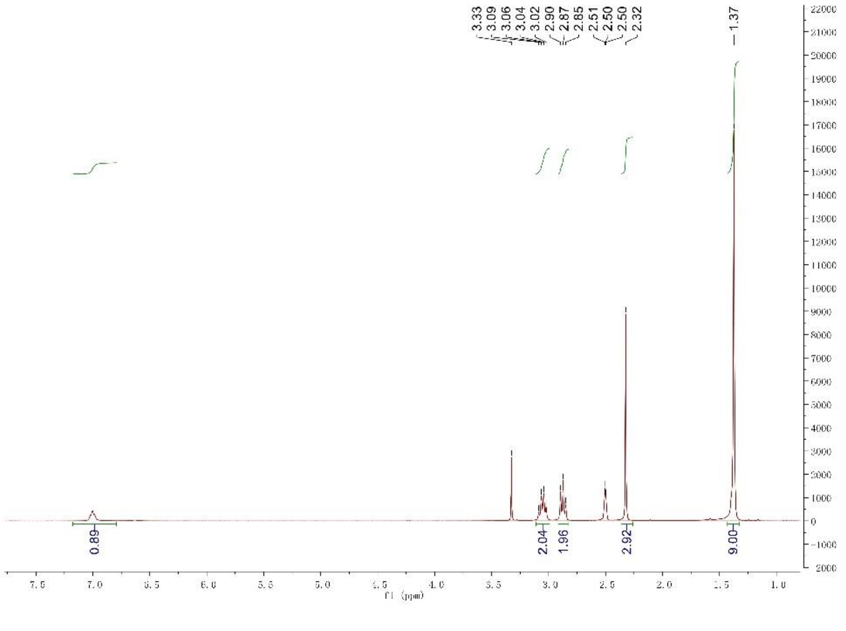

Figure S1. ^{1}H NMR spectrum of compound 3 in DMSO.

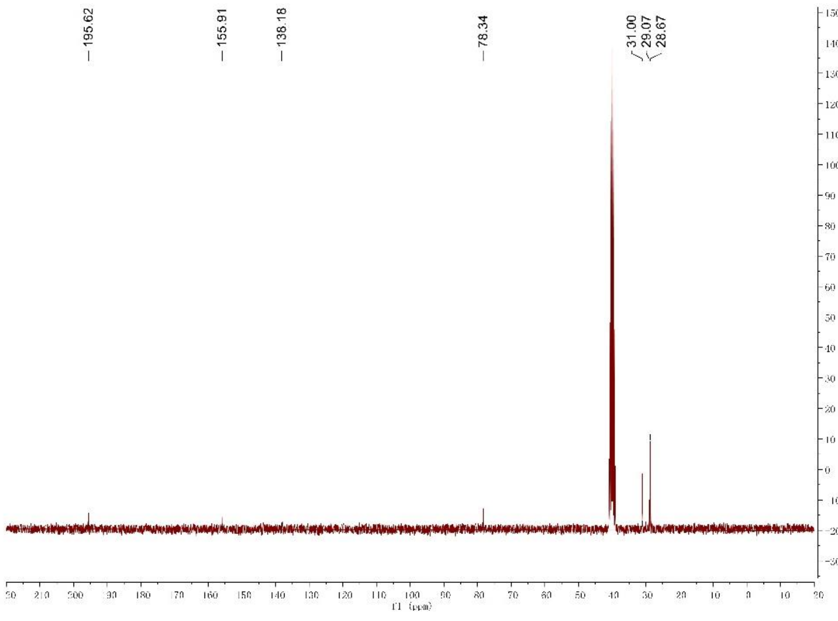

Figure S2. ^{13}C NMR spectrum of compound 3 in DMSO.

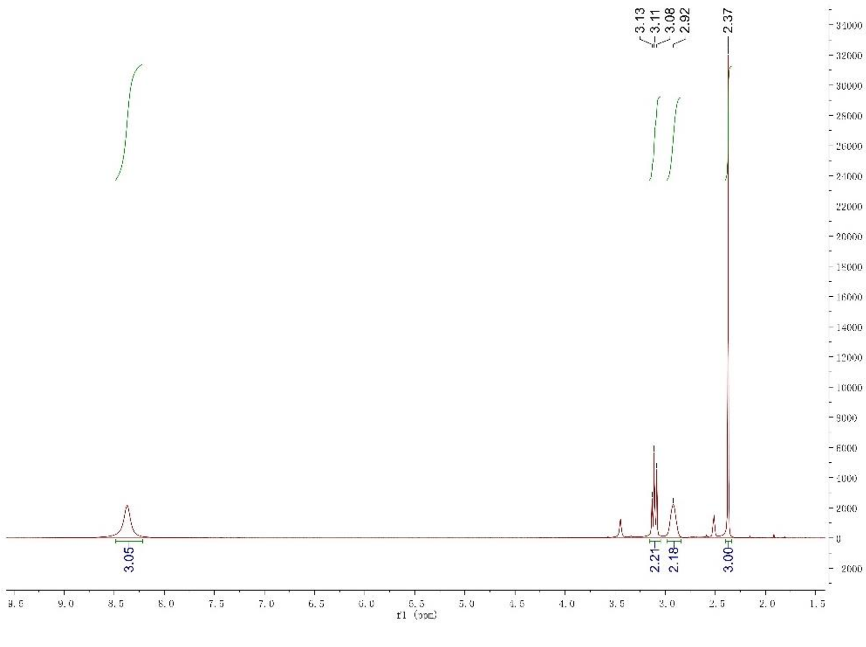

Figure S3. ^{1}H NMR spectrum of compound 2 in DMSO.

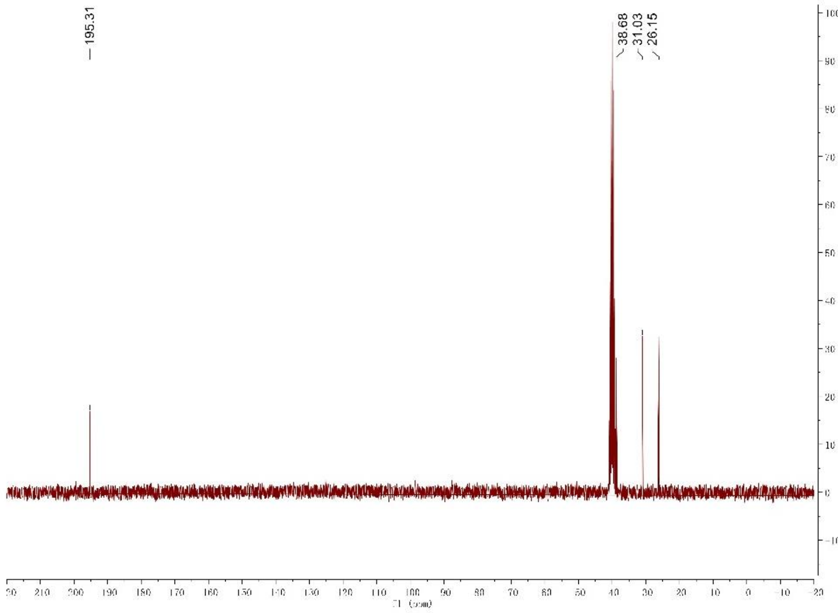

Figure S4. ^{13}C NMR spectrum of compound 2 in DMSO.

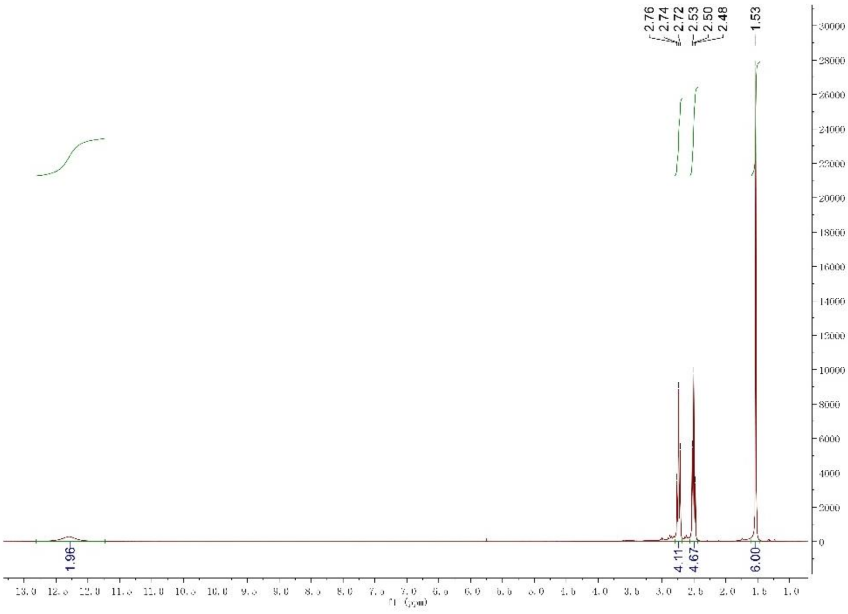

Figure S5. ^{1}H NMR spectrum of TK in DMSO.

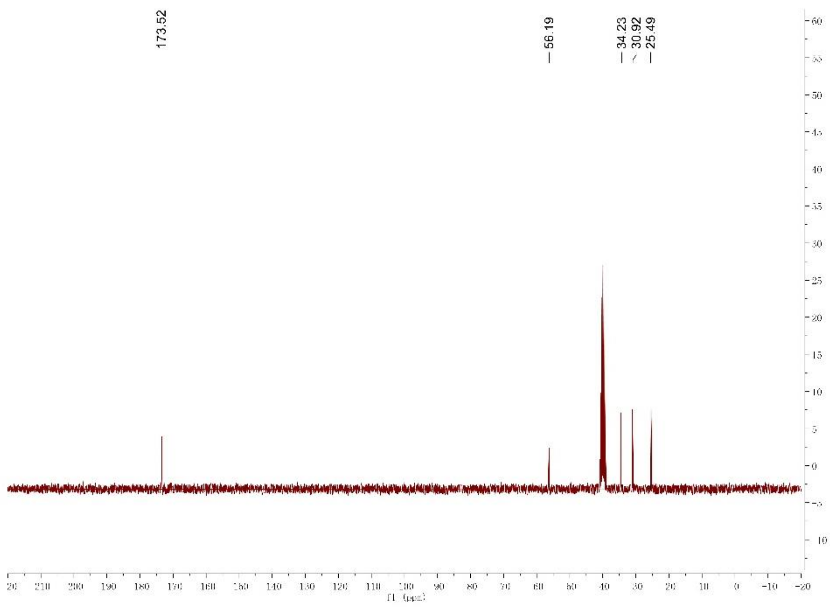

Figure S6. ^{13}C NMR spectrum of TK in DMSO.

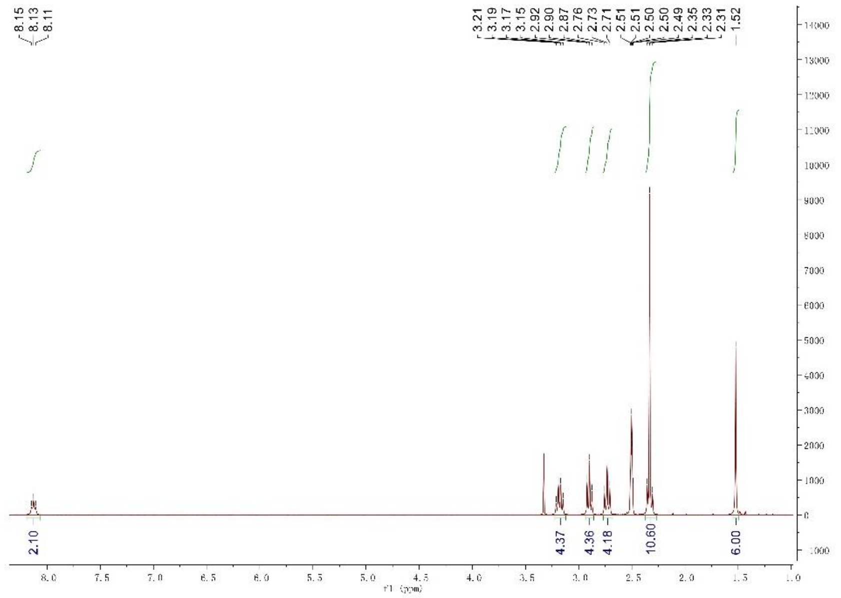

Figure S7. ^{1}H NMR spectrum of M1 in DMSO.

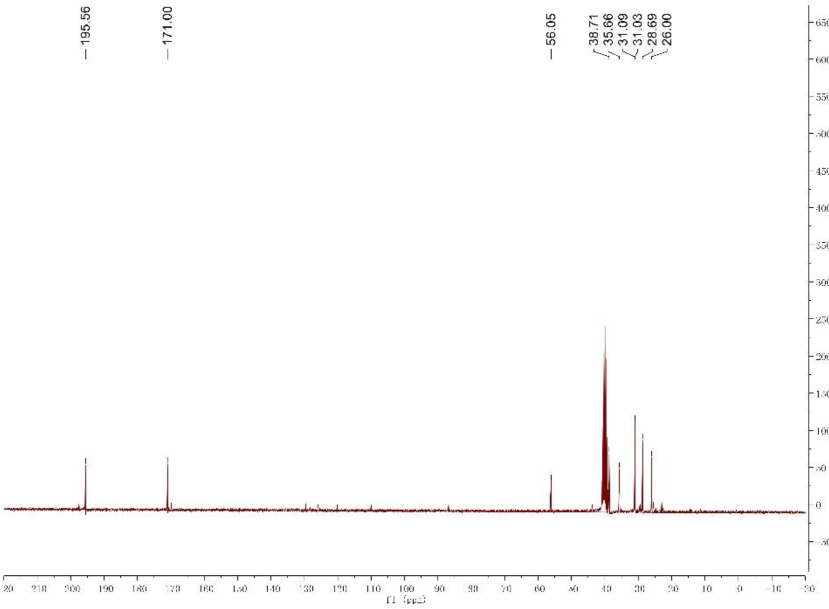

Figure S8. ^{13}C NMR spectrum of M1 in DMSO.

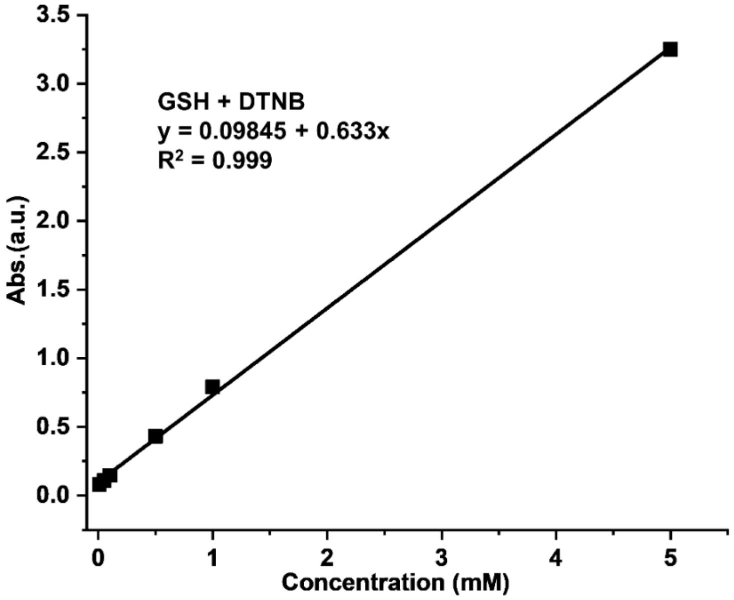

Figure S9. Standard curve between concentrations of glutathione and absorbance at 412 nm.

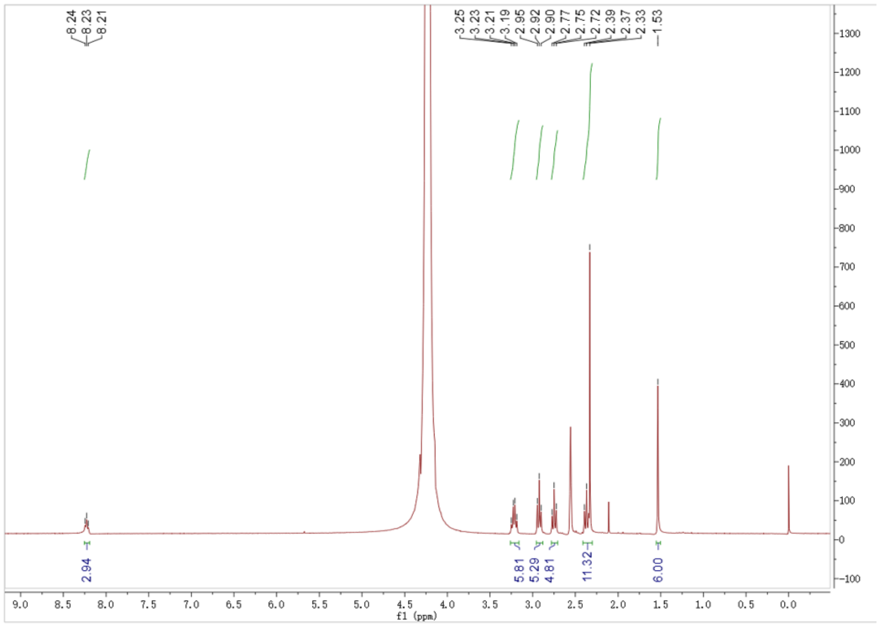

Figure S10. ^{1}H NMR spectrum of M1 with HCl in DMSO.

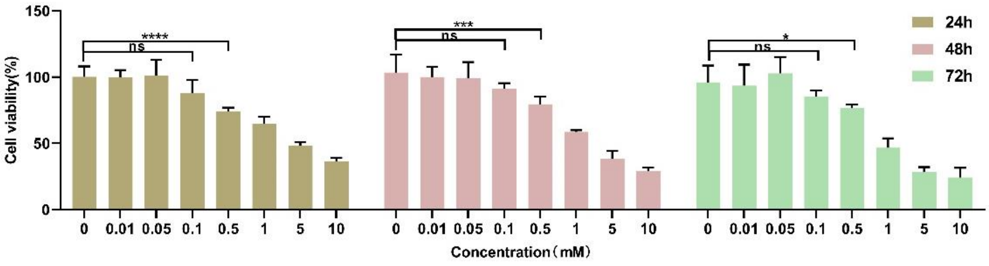

Figure S11. Cytotoxicity assays of M1 with HIEC-6 cells.

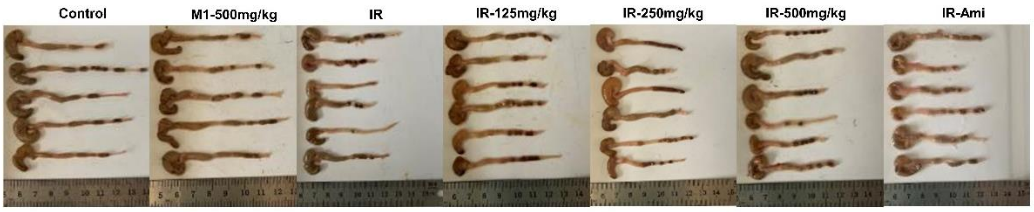

Figure S12. Colon tissues of mice in control, IR, M1-500 mg/kg, IR + 125 mg/kg, IR + 250 mg/kg, IR + 500 mg/kg and IR + Ami groups 3 days after 13 Gy ABI, n = 6 per group.
